# The Calcium Chloride Responsive Type 2C Protein Phosphatases Play Synergistic Roles in Regulating MAPK Pathways in *Magnaporthe oryzae*

**DOI:** 10.3390/jof8121287

**Published:** 2022-12-08

**Authors:** Wilfred M. Anjago, Jules Biregeya, Mingyue Shi, Yixiao Chen, Yupeng Wang, Zonghua Wang, Yonghe Hong, Meilian Chen

**Affiliations:** 1 Ministerial and Provincial Joint Innovation Centre for Safety Production of Cross-Strait Crops, Minjiang University, Fuzhou 350108, China; 2 Ministry of Education Key Laboratory of Biopesticides and Chemical Biology, College of Plant Protection, Fujian Agriculture and Forestry University, Fuzhou 350002, China

**Keywords:** *Magnaporthe oryzae*, protein phosphatase, MAPK signaling pathway

## Abstract

Reversible protein phosphorylation is essential in cellular signal transduction. The rice blast fungus *Magnaporthe oryzae* contains six putative type 2C protein phosphatases, namely MoPtc1, MoPtc2, MoPtc5, MoPtc6, MoPtc7, and MoPtc8. The major functions of MoPtc1 and MoPtc2 have been reported recently. In this communication, we found that MoPtc1 and MoPtc2 were induced by calcium chloride. We also found that the deletion of both MoPtc1 and MoPtc2 resulted in the overstimulation of both the high-osmolarity glycerol (Hog1) and pathogenicity MAP kinase 1 (Pmk1) pathways in *M. oryzae*. MoPtc1 was recruited directly to Osm1 (the osmotic stress-sensitive mutant) by the adaptor protein MoNbp2 to inactivate the Osm1 during hypoosmotic stress, distinct from the budding yeast. Moreover, we showed that MoPtc1 and MoPtc2 were localized in different cellular compartments in the fungal development. Taken together, we added some new findings of type 2C protein phosphatases MoPtc1 and MoPtc2 functions to the current knowledge on the regulation of MAPK signaling pathways in *M. oryzae*.

## 1. Introduction

Type 2C protein phosphatases are monomeric enzymes that exclusively dephosphorylate the serine/threonine residues [1]. In budding yeast, ScPtc1 is a type 2C protein phosphatase that inactivates the osmosensing mechanism of the mitogen-activated protein kinase pathway by dephosphorylating serine or threonine residues of the MAPK-HOG kinase as well as Pbs2 under acute osmolarity [2,3]. According to the results of a yeast two-hybrid assay, Ptc1 interacts with Pbs2 through the adaptor protein Nbp2 in *Saccharomyces cerevisiae* to regulate the HOG pathway [4]. Ptc1-disrupted cells are hypersensitive to LiCl, ZnCl, CaCl_2,_ and rapamycin in *S. cerevisiae* [5]. Among other functions, Ptc1 is also involved in the sporulation, inheritance and distribution of organelles, such as vacuoles, mitochondria and the cortical endoplasmic reticulum, tRNA splicing, cation homeostasis, and the TOR pathway in budding yeast [6,7,8,9]. Ptc2, on the other hand, is crucial in the regulation of the Hog1-MAPK pathway by dephosphorylating the phosphothreonine (pThr) residues of the Hog1 activation loop [10]. A genome search for type 2C protein phosphatases in filamentous fungi such as *Neurospora crassa*, *Fusarium graminearum*, *Botrytis cinerea,* and *Aspergillus* spp. revealed that type 2C encoding genes are widely distributed in all filamentous fungi and six are harboured in the *M. oryzae* genome, suggesting that type 2C protein phosphatases may be involved in major biological and cellular processes, resembling budding and fission yeast [1]. However, their functions in the regulation of MAPK pathways in filamentous fungi are poorly understood.

The hemibiotrophic ascomycete *Magnaporthe oryzae* causes economically devastating blast disease in rice, wheat, and other domesticated grass families [11]. The annual losses resulting from rice blast disease are between 10% to 30%, posing a threat to global food security [12]. The causal agent, *M. oryzae*, is considered a good model for understanding the molecular mechanisms underlying pathogen-host interactions [13]. The disease cycle is initiated after viable conidia tightly adhere to the host surface. The conidium then germinates and differentiates into a dome-shaped cell known as the appressorium in response to physical and chemical cues perceived on the host surface [14,15]. Appressorium development is regulated by multiple signaling pathways involving cAMP and MAPK in *M. oryzae* [16,17]. The highly conserved PMK1-MAPK, a functional homologue of *S. cerevisiae* Fus1/Kss1, is required for the invasive growth and pathogenicity of *M. oryzae* and other appressorium-forming pathogens, such as *Colletotrichum lagenarium* and *Bipolaris oryzae* [18,19], whereas the MAPK-Osm1 pathway is activated in response to hyperosmotic stress, resulting in the accumulation of arabitol and glycerol in rice blast fungus [20,21]. Despite these physiological and pathological defects, the regulation of these MAPK signaling pathways remains elusive in the causative agent of rice blast. It is important to note that the two genes, i.e., MoPtc1 and MoPtc2, have been previously explored [22]. In this study, we not only confirmed the reported results but added some new findings.

In this study, we first evaluated the expression levels of MoPtc1 and MoPtc2 in the presence of multiple stress-inducing agents and found that these genes were significantly expressed by 0.3 M calcium chloride. MoPtc1 and MoPtc2 were further characterized and found to be necessary for regulating the phosphorylation levels of Pmk1-MAPK and Osm1-MAPK in *M. oryzae*. We also found that MoPtc1 was recruited to both MoPmk1 and MoOsm1 by an adaptor protein known as MoNbp2. Furthermore, we showed that the two genes were localized in different cellular compartments in the lifecycle of *Magnaporthe oryzae*.

## 2. Materials and Methods

### 2.1. Fungal Strains and Culture Conditions

*M. oryzae* Guy11 was used as a wild type of strain in this study. The other strains were obtained in this study, named as *∆Moptc1* (Knockout mutant of *MoPtc1* from Guy11), *∆Moptc2* (Knockout mutant of *MoPtc2* from Guy11), *∆Moptc1∆Moptc2* (Double knockout mutant of *MoPtc1* and *MoPtc2*), *∆Moptc1_Com* (Complementation strain of *MoPtc1*), *∆Moptc2_Com* (Complementation strain of *MoPtc2*). The strains were cultured on a complete medium (CM, containing yeast extract 0.6%, casamino acid 0.6%, sucrose 1%, agar 2%) in the 28 °C incubator.

The yeast strain AH109 was cultured in YPDA medium (1% yeast extract, 2% pepton, 2% dextrose, 120 µg/mL adenine hemisulfate) at 200 rpm under 30 °C conditions. Moreover, the transformants of yeast two-hybrid were grown on the SD/-Leu/-Trp media (SD medium without leucine and tryptophan, Clontech, Cat# 630417) and SD/–Ade/–His/–Leu/–Trp media (SD medium without adenine, histidine, leucine and tryptophan, Clontech, Cat# 630428), under 30 °C conditions. Additionally, SD is a synthetically defined medium, supplying everything that a yeast cell needs to survive including carbon, nitrogen sources and essential amino acids.

### 2.2. Quantitative Real Time PCR (qRT-PCR) and RT-PCR Assays

The cells of Guy11, knockout mutants, and complementation strains were shaken in liquid complete media at 130 rpm under 28 °C conditions for 96 h. Total RNA was extracted from the mycelia using the EastepTM Total RNA Extraction Kit (Promega, Madison, WI, USA). Complementary DNA (cDNA) was synthesized using the PrimeScriptTM RT Reagent Kit with a gDNA Eraser (Perfect Real Time). The quantitative real-time PCR (qRT-PCR) was performed using a 10 µL reaction mixture containing 5µL of SYBR^®^ Premix Ex-Taq™, 3.4 µL of DNase/RNase free water, 1µL of 10xcDNA, and 0.3µL of 10µM forward and reverse primers, as recommended by the Promega Corporation Super Real Premix. The qRT-PCR data were produced by the Eppendorf Realplex2 Master Cycler (Eppendorf AG 223341, Hamburg, Germany) and analysis was carried out by using the delta-delta CT (2 −ΔΔCT) method, as previously described [23]. β-tubulin was used as a reference gene, and the primers were designed using the Beacon Designer software (version 8), as shown in Table S1. 

### 2.3. Construction of MoPtc1 and MoPtc2 Complementation Vector with GFP Fusion Proteins 

MoPtc1-GFP and MoPtc2-GFP vectors were constructed by amplifying the 3.5 Kb and 3.9 Kb full-length ORFs of MoPtc1 and MoPtc2 with their respective native promoter regions (the primers were shown in Table S1). The resulting PCR products were cloned into the *Kpn*I/*Hind*III site of pKNTG plasmids with a GFP expression sequence at the C-terminal. The fusion construct was sequenced to validate the orientation of the inserted fragment and transformed into the *∆Moptc1* and *∆Moptc2* protoplasts, respectively. The resulting transformants with G418 resistance were screened by the PCR method with primer pairs MoPTC1 OF-GFP/R and MoPtc2 OF-GFP/R. The GFP signals were screened under Olympus BX51 microscopy (Olympus, Tokyo, Japan) and the pictures were captured with a Nikon A1R laser-scanning confocal microscopy system (Nikon, Tokyo, Japan).

### 2.4. Immunoblotting Assays 

The strains were shaken in complete media (CM) for 72 h and mycelia were ground to fine powder in liquid nitrogen, and then the total protein were extracted and resuspended in 1 mL of protein lysis buffer (10mM Tris-HCl pH-7.5, 150 mM NaCl, 0.5 mM EDTA, and 0.5% Nonidet P-40) in the presence of a 5 µL protease inhibitor cocktail (Sigma-Aldrich. St. Louis, MO, USA) and 1 mM PMSF. Equal amounts (40 µL) of protein were loaded into each well and the total protein was separated on 12.5% SDS-PAGE gels and transferred to nitrocellulose membranes (Amersham, Piscataway, NJ, USA) for Western blot analyses. The membranes were incubated with anti phospho-p38 MAPK (Thr180/Tyr182) (D3F9) XP Rabbit mAb, phospho-p44/42 MAPK (ERK1/2) (Thr202/Tyr204) (D13.14.4E) Rabbit mAb, and p44/42 MAPK (Erk1/2) antibodies (Cell Signaling Technology, Beverly, MA, USA), and affinity purified goat anti-rabbit & mouse antibodies were conjugated to horseradish peroxidase (Abmart, Shanghai, China). The ECL Kit (Amersham Biosciences, Freiburg, Germany) was used to detect the chemiluminescent signals of the specific protein bands [24]. The anti actin (2P2) mouse mAb was used as a reference (Abmart, Shanghai, China).

### 2.5. The Yeast Two-Hybrid Assay

The yeast two-hybrid assay was performed as indicated in the MATCHMAKER GAL4 two-hybrid system 3 (Clontech). The protein coding regions of genes used in this study were amplified from Guy11 cDNA with the primer pairs listed in Table S1. The subsequent cloning of MoPtc1, MoPmk1, and MoOsm1 was performed in the pGBKT7 bait vector, while that of MoNbp1 was performed in the pGADT7 prey vector. The pGBKT7 and pGADT7 cloning vectors were digested with *Nde*I and *Eco*RI restriction enzymes, respectively. These AD and BD constructs pairs were simultaneously co-transformed into the *S. cerevisiae* strain AH109, as previously described [25]. The pGBKT7-53/pGADT7-T and pGBKT7-Lam/pGADT7-T vectors were used as positive and negative controls, respectively. The emerged yeast colonies in the SD/–Leu/–Trp media were isolated and cultured on the SD/-Ade/-His/-Leu/-Trp selective media containing 40μg/mL of X-α-gal (Clontech, Cat# 630463). 

### 2.6. The Co-Immunoprecipitation Assay

For the co-immunoprecipitation assay, GFP fusion proteins from cellular extracts were isolated and incubated with 20–30 μL of GFP-Trap magnetic beads (ChromoTek, Martinsried, Germany), according to the manufacturer’s instructions. Proteins eluted from GFP-Trap magnetic beads were analyzed following immunoblot detection with the anti-Flag (Abmart) and anti-GFP (Abmart) antibodies. To elute the proteins from GFP-Trap magnetic beads, we first placed the 10 mL tubes in ice for 10 min for magnetic beads to settle at the bottom. Then, the tubes were transferred to a magnetic rack, and then we carefully removed the supernatant. After washing three times with 1mL washing buffer (50mM Tris, 150mM NaCl, pH7.4), the beads were resuspended and boiled with 80 µL washing buffer and 20 µL 5× SDS loading buffer. At last, 40 µL of the total protein was loaded into 10% SDS-PAGE gel for western blotting and co-immunoprecipitation analysis.

### 2.7. Statistical Analysis

Statistical analysis was performed using Graph Pad Prism 7 and the data from the independent biological replicates were analyzed with a one-way ANOVA (and non-parametric) and the Dunnett’s multiple-comparison test with a 0.05 significance level (95% confidence interval).

## 3. Results and Discussion

Through a protein blast search (BLASTP) using *S. cerevisiae* type2C protein phosphatases amino acid sequences as protein queries, we identified five single PP2C orthologs in the genome of *M. oryzae,* that included MoPtc1, MoPtc2, MoPtc5, MoPtc6 and MoPtc7. The new member MoPtc8 was not encoded in *S. cerevisiae* genome; hence, it was identified in *M. oryzae* via a protein blast search (BLASTP) using *C. albicans* CaPtc8 amino acid sequences. Among the six members, *MGG_05207* and *MGG_01351* encoded for MoPTC1 and MoPTC2, respectively. The primary structures and percentage identities with *S. cerevisiae* counterparts were described in Table 1.

An examination of the domain architecture with the SMART database (accessed on 10 September 2020, http://smart.embl-heidelberg.de/) revealed that MoPtc1 contains a PP2Cc (serine/threonine phosphatase, family 2C, catalytic) domain at amino acid positions 141-478 and a PP2C_SIG (sigma factor PP2C-like phosphatase) domain at amino acid positions 190-480 (Figure S1). MoPtc2, on the other hand, is a protein with a length of 461 amino acids that contains a PP2Cc domain at position 13-293 and a PP2C_SIG domain at position 39-295 (Figure S1).

**Figure 1:**
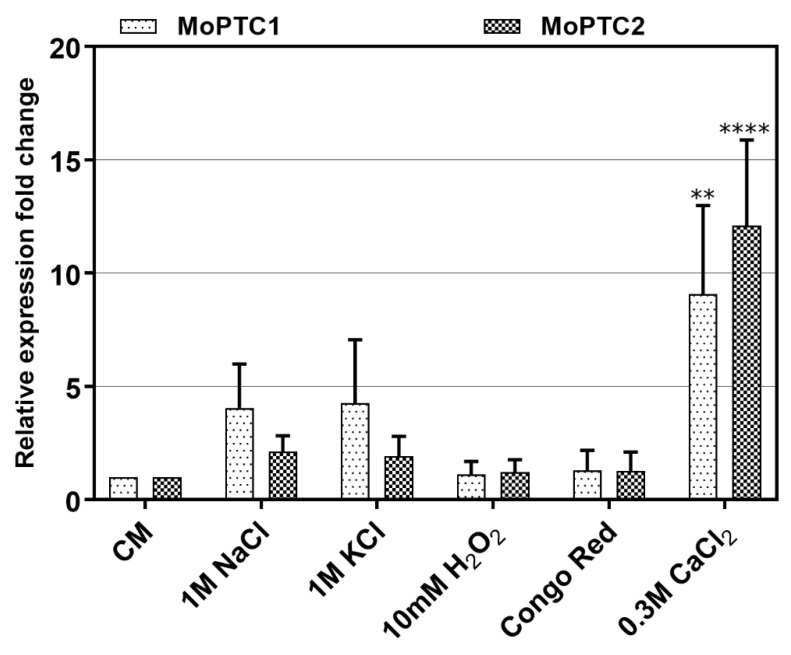
Transcript expression patterns of MoPTC1 and MoPTC2 in different stress-causing agents. In this assay, β-tubulin was used as the reference gene. The error bars represent the mean standard error from three independent replicates. The double asterisks indicate the adjusted *p*-value of 0.0013 and the quadruple asterisks show the adjusted *p*-value of <0.0001.

To monitor the expression levels of type 2C protein phosphatases-encoding genes, we first conducted the quantitative real-time PCR (qRT-PCR) during pathogen-host interactions. Three-week-old susceptible rice seedlings were sprayed with Guy11 spore suspensions and RNA was extracted from these infected plant tissues at different time intervals. From the results, we confirmed that MoPtc1 and MoPtc2 were expressed at different developmental stages of the infectious cycle and showed significant expression at 12h post-incubation as the recently reported [22] (Figure S2). These findings suggest that MoPtc1 and MoPtc2 may play important roles in appressorium development in *M. oryzae*. Next, we cultured Guy11 wild type strain in the CM media with or without different stress inducers and quantified the transcript abundance of MoPtc1 and MoPtc2. The results showed that MoPtc1 and MoPtc2 were differentially expressed in osmotic (NaCl and KCl), and ionic (CaCl_2_) stressors, and the highest transcript expression was found when upon exposure to the ionic stress induced by CaCl_2_ (Figure 1). These results support the idea that MoPtc1 and MoPtc2 are involved in the regulation of calcium homeostasis in *M. oryzae*.

To reveal the functions of MoPtc1 and MoPtc2 in *M. oryzae*, the gene deletion construct for each studied gene was amplified with a double-joint PCR and transformed into protoplasts of the wild-type strain Guy11. The obtained transformants were screened by the PCR, and the resulting mutants were confirmed by Southern blotting (Figure S3). For each gene, only one mutant was used for further analysis. The genetic complementation of *∆Moptc1* and *∆Moptc2* mutants was performed by transforming MoPtc1-GFP and MoPtc2-GFP under the control of their native promoters into the deletion mutants. The primers used in the study are listed in Table S1.

**Figure 2:**
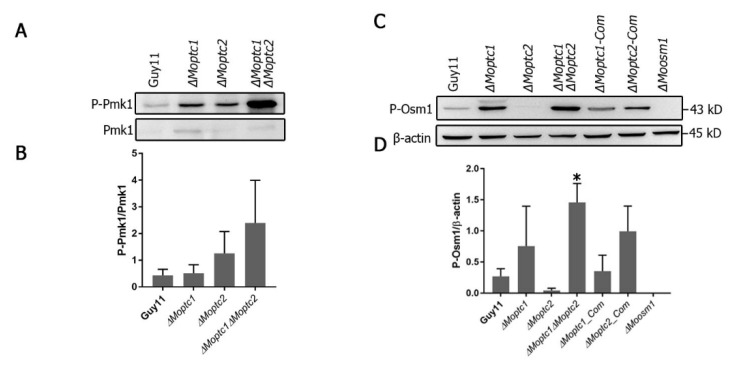
MoPtc1 and MoPtc2 regulate MAPK signaling cascades in *M. oryzae*. (**A**) The Western blot assay shows the Pmk1 phosphorylation level in Guy11, *∆Moptc1*, and *∆Moptc2* and *∆Moptc1∆Moptc2* strains. (**B**) The extent of Pmk1 phosphorylation was estimated by calculating the amount of phosphorylated Pmk1 (P-Pmk1) compared to the total amount of Pmk1. (**C**) Western blot images quantify the Osm1-MAPK phosphorylation in Guy11 and *∆Mopt*c1, *∆Moptc2*, *∆Moptc1∆Moptc2*, *∆Moptc1_Com* (Complementation), *∆Moptc2_Com* (Complementation), and *∆Moosm1*. (**D**) The band intensity ratio between phosphorylated Osm1 (P-Osm1) and β-actin. The single asterisk (*) represents statistical significance with an adjusted *p*-value of 0.0338.

Western blot analysis, used to assay the phosphorylation level of MoPmk1 and MoOsm1, revealed the role of MoPtc1 and MoPtc2 in the regulation of the MAPK signaling pathway. The phosphorylation levels of Pmk1 were increased in both *∆Moptc1* and *∆Moptc2* single deletion mutants and *∆Moptc1∆Moptc2* double deletion strains compared to Guy11 (Figure 2A,B). For testing the phosphorylation level of Osm1, the total protein was extracted from the mycelia of Guy11 wild type strain, *∆Moptc1*, *∆Moptc2*, *∆Moptc1∆Moptc2*, *∆Moptc1*_Com, and *∆Moptc2*_Com strains. An increase in Osm1 phosphorylation was detected in *∆Moptc1* and *∆Moptc1∆Moptc2* mutants, while the Osm1 phosphorylation levels was decreased in *Moptc2* mutant (Figure 2C,D). We also noted an increase in the phosphorylation levels in MoPtc2 complementation strain, suggesting that the decreased effect in Osm1 phosphorylation was caused by the deletion of MoPtc2. These results strongly suggest that MoPtc1 and MoPtc2 played synergistic roles in dephosphorylating Pmk1-MAPK in *M. oryzae*. In addition, MoPtc1 was found to be a negative regulator of Osm1 during hypoosmotic stress, while MoPtc2 positively regulated this pathway. 

**Figure 3:**
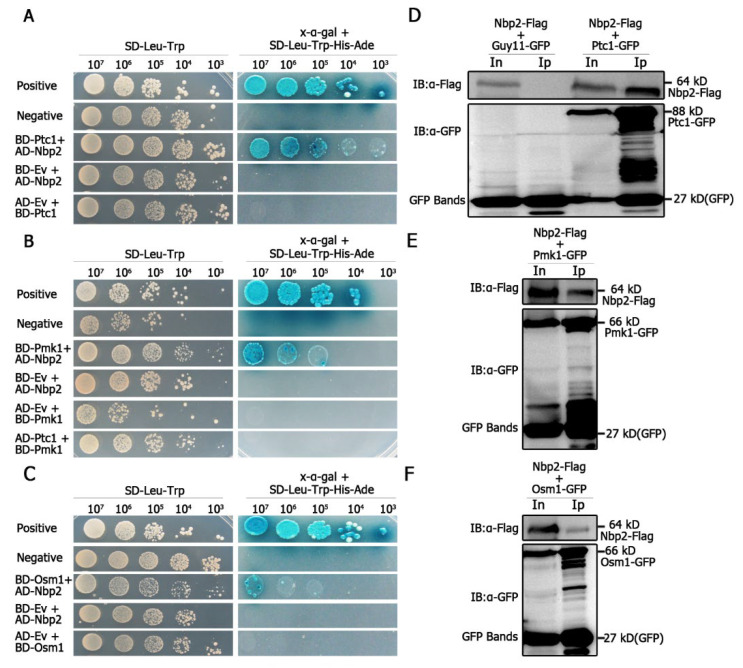
MoPtc1 interacts with MoPmk1 and MoOsm1 in *M. oryzae*. The yeast two-hybrid assay shows the interactions between (**A**) MoPtc1 and MoNbp2, between (**B**) MoNbp2 and MoPmk1, and between (**C**) MoNbp2 and MoOsm1 in rice blast fungus. The pGBKT7-53/pGADT7-T and pGBKT7-Lam/pGADT7-T vectors were used as positive and negative controls, respectively. The co-transformation of prey and bait constructs into the yeast strain resulted in the transcription activation of reporter genes and growth on the selective medium -Leu/-Trp/-His/-Ade. The yeast colonies turned blue after the secretion of β-galactosidase (LacZ) in the selective medium and the hydrolysis of X-α-gal. The co-immunoprecipitation assay confirms the interactions between (**D**) MoPtc1 and MoNbp2, between (**E**) MoNbp2 and MoPmk1, and between (**F**) MoNbp2 and MoOsm1 in *M. oryzae*.

We assayed for the possible interaction between MoPtc1 or MoPtc2 with Pmk1 or Osm1 in *M. oryzae* via yeast two-hybrid assays. The results showed a strong interaction between MoPtc1 and the adaptor protein MoNbp2 (Figure 3A), and between MoNbp2 and Pmk1 (Figure 3B), and between MoNbp2 and MoOsm1 (Figure 3C) in *M. oryzae*. These interactions were further confirmed by the co-immunoprecipitation (Co-ip) assay in vivo. First, we co-transformed Nbp2-Flag into the protoplast of Guy11-GFP as our control experiment and were able to detect a 64 kD band size of Nbp2 using an anti-Flag antibody, as well as a GFP band size of 27 kD using an anti-GFP antibody (Figure 3D). Secondly, we co-transformed Nbp2-Flag and Ptc1-GFP constructs into Guy11 protoplasts and were able to detect an 88 kD band size corresponding to Ptc1-GFP and a 64kD band size for Nbp2-Flag in the total protein and GFP magnetic beads (Figure 3D). Thirdly, we co-transformed Pmk1-GFP and Nbp2-Flag constructs into Guy11 protoplasts and were able to detect both the Pmk1-GFP (66 kD) band and the Nbp2-Flag (64 kD) band in the total proteins and co-immunoprecipitation lysates (Figure 3E). Finally, we co-transformed Osm1-GFP and Nbp2-Flag constructs into Guy11 protoplasts and found that the anti-Flag antibody was able to specifically detect a clear band of 64 kD, while the anti-GFP antibody was able to detect a clear band of 66 kD (Figure 3F). Together these results demonstrate that MoPtc1 interacts with Pmk1 and Osm1 via the adaptor protein in *M. oryzae*. This is the first report in our knowledge to explore the regulation of MAPK signaling pathways through adaptor proteins in plant pathogenic fungi.

**Figure 4:**
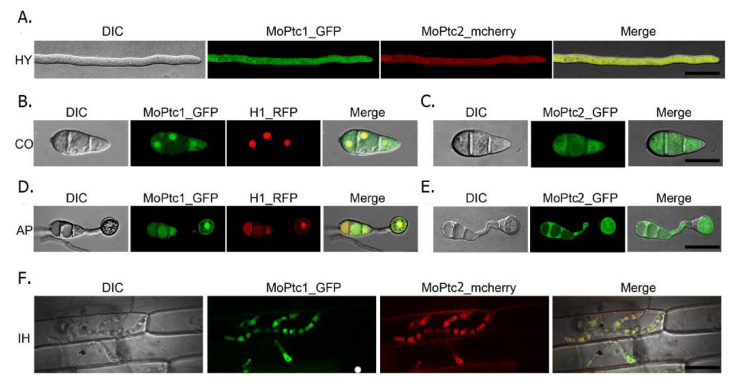
The subcellular localization of MoPtc1 and MoPtc2 protein phosphatases in *M. oryzae*. (**A**) The colocalization of MoPtc1 and MoPtc2 in hyphal (HY) stages of development (**B**) The localization pattern of MoPtc1 in conidia (CO). (**C**) The localization of MoPtc2 in conidia (CO). (**D**,**E**) The localization of MoPtc1 and MoPtc2 in appressorium (AP) development. (**F**) The colocalization of MoPtc1 and MoPtc2 in invasive hypha (IH). H1_RFP is the Histone 1 fused with red fluorescence protein. The live cell images were visualized by Nikon Air Laser confocal microscopy (scale bar = 20 µm).

To determine the subcellular localization of MoPtc1 and MoPtc2, we firstly co-transformed MoPtc1-GFP and MoPtc2-mcherry with a constitutive promoter in Guy11 protoplasts and found that MoPtc1 was co-localized with MoPtc2 in the cytoplasm in mycelium (Figure 4A) and invasive hypha (Figure 4F). We also checked for the localization of MoPtc1 and MoPtc2 by transforming the MoPtc1-GFP, and MoPtc2-GFP, under a constitutive promoter into the protoplasts of Guy11 and found that MoPtc1 was localized in the nucleus and cytoplasm in both conidia (Figure 4B) and appressorium (Figure 4D) stages of development, while MoPtc2 was localized in the cytoplasm (Figure 4C,E). Taken together, our results demonstrate that type 2C protein phosphatases MoPtc1 and MoPtc2 were localized in different cellular compartments in the life cycle of *M. oryzae*.

**Figure 5:**
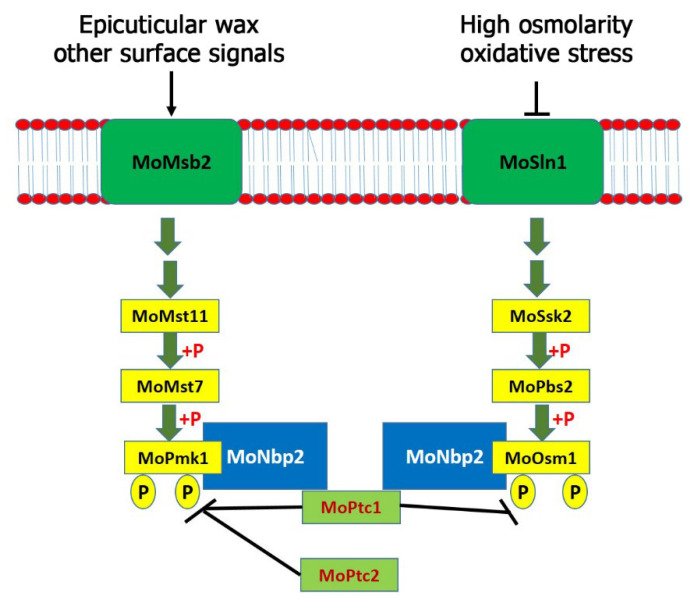
A model showing regulation of MAPK signaling pathways by MoPtc1 and MoPtc2 in *M. oryzae*. The Pmk1 pathway is essential for appressorium formation and invasive growth in this fungus. We identified MoPtc1 and MoPtc2 as negative regulators of the MoPmk1-MAPK in *M. oryzae*. The Osm1 pathway was activated during high osmolarity and oxidative stress and played crucial role in osmotic stress response. This pathway was negatively regulated by MoPtc1 and positively regulated by MoPtc2. The MoPtc1 interacted with MoOsm1-MAPK through the adaptor protein MoNbp2 both in vitro and in vivo.

In conclusion, the type 2C protein phosphatases MoPtc1 and MoPtc2 were significantly and differentially upregulated by 0.3M CaCl_2_. These results showed that both proteins may be involved in the ionic stress response in *M. oryzae*. Additionally, MoPtc1 and MoPtc2 play synergistic roles in negatively regulating the MoPmk1. Similar to the budding yeast, MoPtc1 strongly interacted with MoNbp2; however, MoNbp2 interacted with MoOsm1 and MoPmk1 (but not MoPbs2) in *M. oryzae* as shown in the model (Figure 5). Three MAPK genes were identified and shown to regulate various developmental processes in *M. oryzae*. The Pmk1 pathway is essential for appressorium formation and invasive growth in this fungus. We have identified MoPtc1 and MoPtc2 as negative regulators of the MoPmk1-MAPK in *M. oryzae*. Distinct from the budding yeast, MoPtc1 was found to interact with MoPmk1 via the adaptor protein MoNbp2, both in vitro and in vivo. The deletion of both MoPtc1 and MoPtc2 resulted in increased phosphorylation levels of MoPmk1 in this fungus, suggesting that the two proteins play a synergistic role in regulating the phosphorylation levels of Pmk1. The Osm1 pathway was activated during high osmolarity and oxidative stress and played crucial role in osmotic stress response. This pathway was found to be negatively regulated by MoPtc1 and positively regulated by MoPtc2. In contrast, Ptc1 and Ptc2 were reported as negative regulators of the Hog1 pathway in the budding yeast [10]. We detected an increase in the Osm1 phosphorylation levels in the MoPtc1 deletion mutant and a decrease in the Osm1 phosphorylation levels in the MoPtc2 deletion mutant. The MoPtc1 interacted with MoOsm1-MAPK through the adaptor protein MoNbp2, both in vitro and in vivo. In summary, MoPtc1 was recruited to MoPmk1 and MoOsm1 via the adaptor protein MoNbp2 in *M. oryzae*. However, it is difficult to tell the possible causes of the different roles of MoPtc1 and MoPtc2 in the Osm1 phosphorylation compared with the roles of both Ptc proteins on Pmk1 phosphorylation because they are two different pathways which are activated by different environmental or cellular conditions. Finally, we showed that MoPtc1 and MoPtc2 were localized in different cellular compartments in the life cycle of *M. oryzae*. Understanding the regulatory mechanisms of MAPK signaling pathways could lead to the discovery of new anti-blast agents that will prevent the spread of rice blast disease. 

## Figures and Tables

**Table 1:** Primary structure analysis of six type 2C protein phosphatases in *M. oryzae*.

Gene	Accession Number in NCBI and Fungi Database	The Nucleotide Sequence Length Including Introns (bp)	Protein Length Based on Amino Acid Sequence (aa)	The Amino Acid Sequence Identity to *S. cerevisiae* Type 2C Phosphatases
*MoPTC1*	XP_003712752	3752	561	16.20% to ScPtc1
*MoPTC2*	XP_003714280.1	3364	451	34.70% to ScPtc3
*MoPTC* *5*	XP_003716839.1	2494	620	33.70% to ScPtc5
*MoPTC* *6*	XP_003719948.1	2470	696	11.60% to ScPtc6
*MoPTC* *7*	XP_003718959.1	1104	367	18.50% to ScPtc7
*MoPTC* *8*	XP_003713735.1	2039	432	*-*

## Data Availability

The data that support the findings of this study are available from the corresponding author upon reasonable request.

## Supplementary Materials

The following supporting information can be downloaded at: https://www.mdpi.com/article/10.3390/jof8121287/s1, Table S1: A list of primers used in this study; Figure S1: Domain architecture of type 2C protein phosphatases in *Magnaporthe oryzae*; Figure S2: In planta expression pattern of MoPTC1 and MoPTC2, at 4, 8, 12, 24, 48 and 72 h of infection; Figure S3: Targeted gene deletion for *∆Moptc1*, *∆Moptc2*, and *∆Moptc1∆Moptc2* in *M. oryzae.*
