# Supplementary material for: The Calcium Chloride Responsive Type 2C Protein Phosphatases Play Synergistic Roles in Regulating MAPK Pathways in Magnaporthe oryzae
Source: J Fungi (Basel). 2022 Dec 8;8(12):1287. doi: 10.3390/jof8121287 (PMC9784850; doi:10.3390/jof8121287)
Supplement: Supplementary file 1 [file jof-08-01287-s001.zip › jof-2031212-supplementary.pdf]

Supplementary:

Table S1. A list of primers used in this study.

Name	Sequence 5' to 3'	Purpose
MGG_05207 AF	GCCAAAATGAGATACCAGAC	To amplify A fragment for MoPtc1 deletion
MGG_05207 AR	AGGGAACAAAAGCTGGGTACCCAGAGACGGTTGCAGAGACAC	
MGG_05207 BF	GAATAGAGTAGATGCCGACCGCGGGTTGAGAAACAGCCCGCATAG	To amplify B fragment for MoPtc1 deletion
MGG_05207 BR	TTCAACGACCACGAAAGC	
MGG_05207 OF	AAAACCACAGCCACTCCG	To amplify ORF fragment for MoPtc1 deletion
MGG_05207 OR	CGCTTGCTTGTCAAATCG	
MGG_05207 UA	CGGTCGGTGGCGGTAGTGAT	To amplify UAH fragment for testing MoPtc1 deletion mutants
H853	GACAGACGTCGCGGTGAGTT	
MGG_01351 AF	TTGTGATTCTGTCGGTTC	To amplify A fragment for MoPtc2 deletion
MGG_01351 AR	TTGACCTCCACTAGCTCCAGCCAAGCCTTACGGTTGACTCCTGAG	
MGG_01351 BF	GAATAGAGTAGATGCCGACCGCGGGTTTCCCCTACACCTTTGACCT	To amplify B fragment forMoPtc2 deletion
MGG_01351 BR	GCAATCTGAATCTCGTCCC	
MGG_01351 OF	GGTGATGATGATGAGTTCT	To amplify ORF fragment for MoPtc2 deletion
MGG_01351 OR	ATCTTTGGTCCCTTTGTC	
MGG_01351 UA	CATCTTTCCGAGGTGGCG	To amplify UAH fragment for testing MoPtc2 deletion mutants
H853	GACAGACGTCGCGGTGAGTT	
MGG_05207 Com-F	GAACAAAAGCTGGGTGAGAGGAGGCGCGTTTT	To amplify Ptc1 complementation fragment

MGG_05207 Com-R	CTGCAGGCATGCAAGTTGAAGATGTGGCCGGTT	
MGG_01351 Com-F	GAACAAAAGCTGGGTGTCGCAATACTCGGTCTT	To amplify Ptc2 complementation fragment
MGG_01351 Com-R	CTGCAGGCATGCAAGGACCTTGATATCCTCGT	
MoPTC1 AD-F	GTACCAGATTACGCTCATATGATGTTTGGCGGCTCCTC	For making Ptc1-AD construct of yeast two hybrid
MoPTC1 AD-R	ATGCCCACCCGGGTGGAATTCTTATGAAGATGTGGCCGGTT	
MoPTC1 BD-F	TCAGAGGAGGACCTGCATATGATGTTTGGCGGCTCCTC	For making Ptc1-BD construct of yeast two hybrid
MoPTC1 BD-R	TCGACGGATCCCCGGGAATTCTTATGAAGATGTGGCCGGTT	
MoNBP1 AD-F	GTACCAGATTACGCTCATATGATGTCTCGCGCCAATCC	For making Nbp1-AD construct of yeast two hybrid
MoNBP1 AD-R	ATGCCCACCCGGGTGGAATTCTTACCGCATAATTTCCTGG	
MoPMK1 BD-F	TCAGAGGAGGACCTGCATATGATGTCTCGCGCCAATCC	For making Pmk1-BD construct of yeast two hybrid
MoPMK1 BD-R	TCGACGGATCCCCGGGAATTCTTACCGCATAATTTCCTGG	
MoOSM1 AD-F	GTACCAGATTACGCTCATATGATGGCGGAATTCGTGCG	For making Osm1-AD construct of yeast two hybrid
MoOSM1 AD-R	ATGCCCACCCGGGTGGAATTCTTATTGGCCGGTAAACT	
MoOSM1 BD-F	TCAGAGGAGGACCTGCATATGATGGCGGAATTCGTGCG	For making Osm1-BD construct of yeast two hybrid
MoOSM1 BD-R	TCGACGGATCCCCGGGAATTCTTATTGGCCGGTAAACT	
HG-F	GAATAGAGTAGATGCCGACCGCGGGTT	To amplify AH fragment for gene deletion
HG-R	TTGACCTCCACTAGCTCCAGCCAAGCC	To amplify HB fragment for gene deletion
MGG_05207 A2F	GCCAAAATGAGATACCAGAC	To amplify A fragment for double knocking out

MGG_0520<br>7 A2R	GGAAATTGTAAGCGTTAATCTAGAGCG<br>CGGTTGCAGAGACAC	
MGG_0520<br>7 BF	GCATTCTGGGTAAACGACTCATAGGAG<br>GAGAAACAGCCCGCATAG	To amplify A fragment for double knocking out
MGG_0520<br>7 BR	TTCAACGACCACGAAAGC	
MoPTC1<br>QF	CATCCCTATACGACCGAAACTG	For quantitative real-time PCR
MoPTC1<br>QR	CCGCTGGGTCTTCGATATTT	
MoPTC2<br>QF	CGACAGAGGACCAGACAAATAA	For quantitative real-time PCR
MoPTC2<br>QR	TCACAGCAGCGTCAATGT	
Tubulin QF	TCGACAGCAATGGAGTTTAC	For quantitative real-time PCR (as a internal reference)
Tubulin QR	AGCACCAGACTGACCGAAGAC	

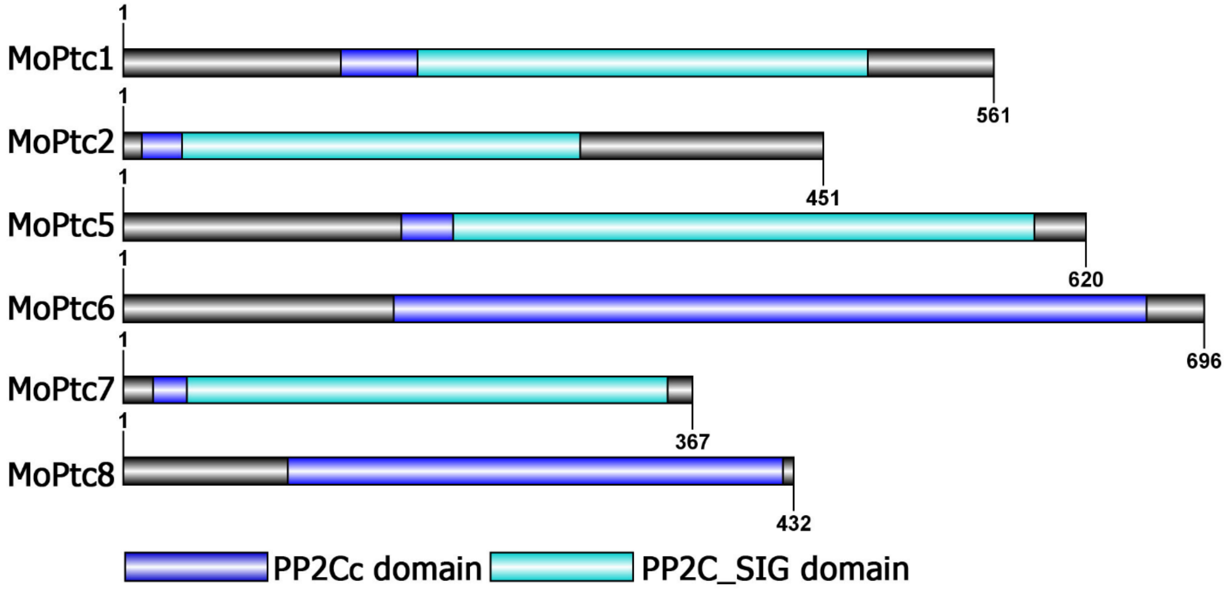

Figure S1. Domain architecture of type 2C protein phosphatases in *Magnaporthe oryzae*.

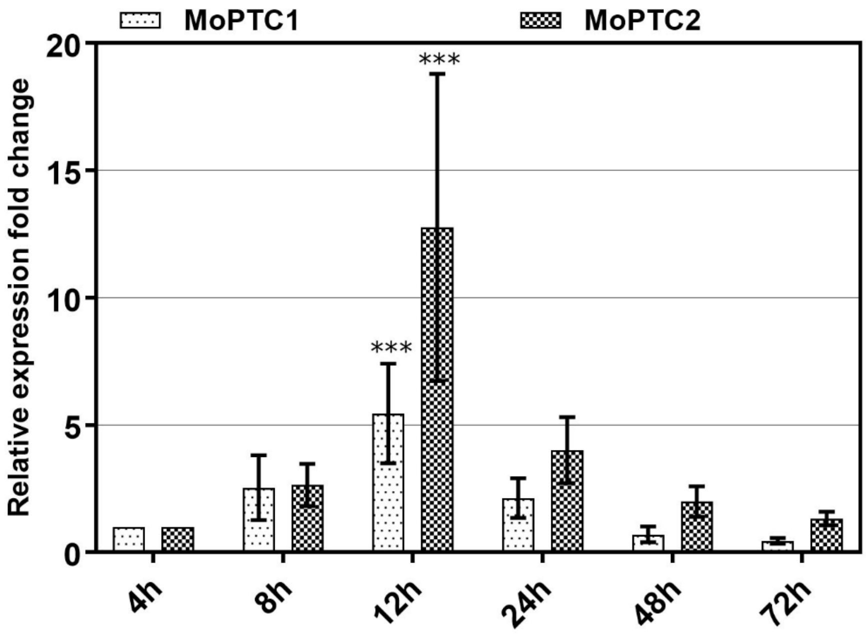

Figure S2. In planta expression pattern of MoPTC1 and MoPTC2, at 4, 8, 12, 24, 48 and 72 h of infection. The triple asterisks denotes adjusted *p* value of 0.0007.

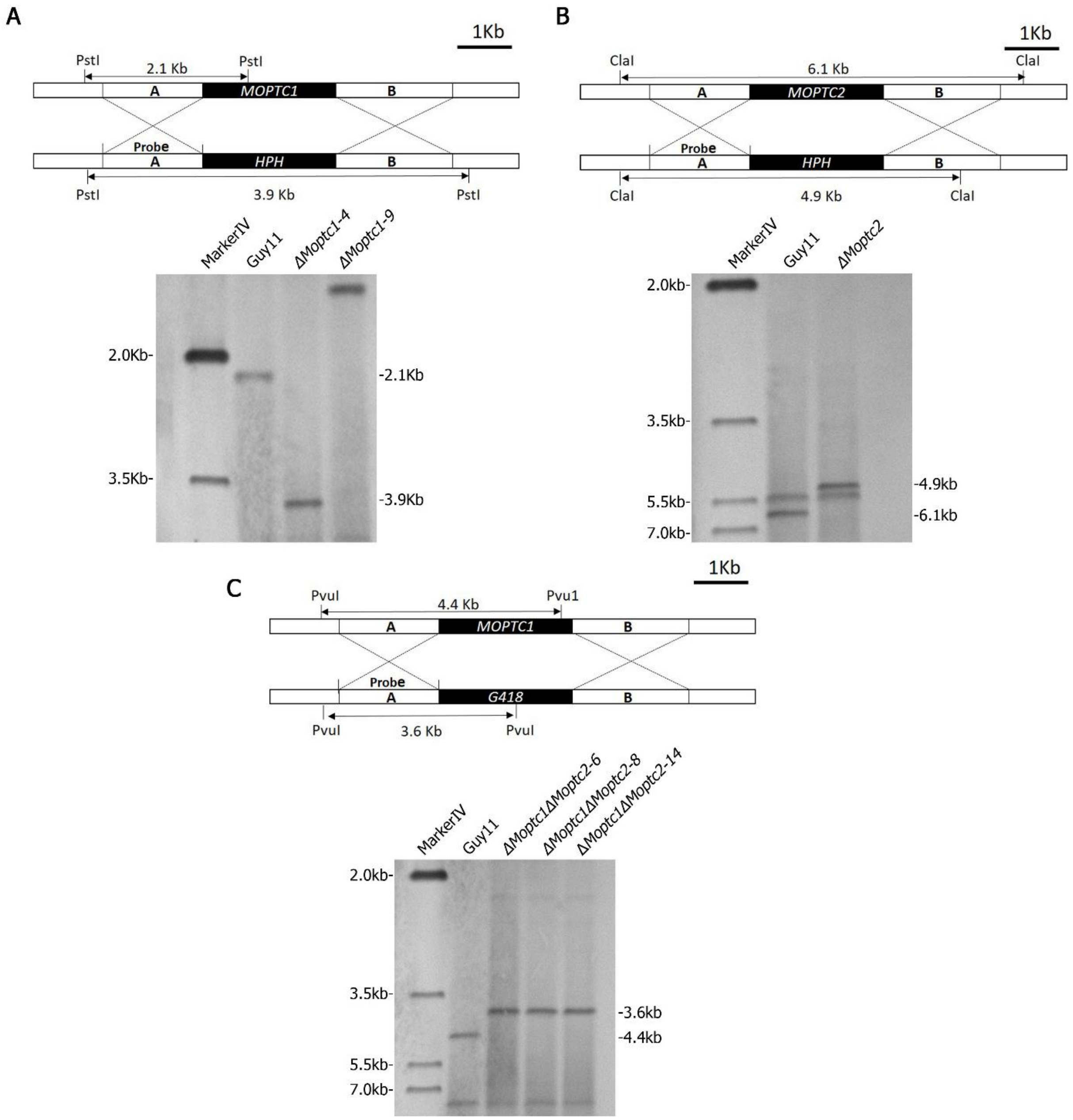

Figure S3. Targeted gene deletion for Δ*Moptc1*, Δ*Moptc2*, and Δ*Moptc1*Δ*Moptc2* in *M. oryzae.*
